# Youth coping and cardiac autonomic functioning: Implications for social and academic adjustment

**DOI:** 10.1002/dev.22338

**Published:** 2022-11-23

**Authors:** Xiaomei Li, Tianying Cai, Virnaliz Jimenez, Kelly M. Tu

**Affiliations:** ^1^ Department of Human Development and Family Studies University of Illinois at Urbana–Champaign Urbana Illinois USA

**Keywords:** academic adjustment, autonomic nervous system, cardiac autonomic regulation, coping, early adolescence, peer adjustment

## Abstract

Extending literature on youth coping and stress physiology, this two‐wave longitudinal study examined independent and interactive roles of youth coping with daily stressors (i.e., peer, academic) and cardiac autonomic functioning in subsequent social and academic adjustment across the transition to middle school. Our sample consisted of 100 typically developing youth (10–12 years old at Time 1, 53 boys, 43% ethnic minorities) who reported on their coping strategies in response to peer and academic stress. Youth participated in laboratory tasks (i.e., baseline, mother–youth conversations about youth's actual peer and academic challenges) during which sympathetic and parasympathetic activities were recorded, and cardiac autonomic functioning indicators were derived. Youth, mothers, and teachers reported on various aspects of youths’ social and academic adjustment at Times 1 and 2. Results revealed that, for both peer and academic domains, greater use of engagement coping strategies was prospectively linked with better adjustment 7 months later, but only among youth who exhibited higher (greater sympathetic–parasympathetic coactivation) but not lower (limited coactivation, or coinhibition) cardiac autonomic regulation at baseline. Findings suggest that a match between more engagement coping behaviors and greater cardiac autonomic capacity to coactivate the parasympathetic and sympathetic branches is linked with better social and academic adjustment.

## 1 INTRODUCTION

Youth face prominent demands in the peer and academic domains during the transition to middle school. Entry into middle school is often accompanied by considerable changes in youths’ social circles, including larger and more diverse peer groups, increased time and intimacy with friends, greater need for affiliation, as well as greater attention to social reputation (Juvonen et al., 2004; Rubin et al., 2011; Westenberg et al., 2004). Changing peer dynamics can be significant sources of stress for youth, and difficulties adapting to these changes can confer risk for poor social adjustment, including negative relationships with peers (e.g., low friendship quality, low social status), more peer problems (e.g., rejection, victimization), and difficulties in developing adaptive social skills (for reviews, see Erath & Pettit, 2021; Shin & Ryan, 2012). Additionally, changes in the academic domain such as differences in school structure, increasing academic demands, new relationships with teachers, and higher expectations for academic achievement pose challenges to youths’ academic success (e.g., subject performance, school engagement, learning motivation; Goldstein et al., 2015; Rudolph et al., 2001). Given that poor social and academic outcomes are intertwined with more general psychopathology and behavioral maladjustment (e.g., depression, social anxiety, aggression, low self‐esteem; Hawker & Boulton, 2000; Liu et al., 2018; Suldo & Shaffer, 2008), understanding how youth respond to these common, yet salient peer and academic stressors and the implications for social and academic adjustment can shed light on adolescent well‐being during this transitional period.

Prior work informs two distinct but interrelated facets of youth stress response—voluntary and involuntary (Compas et al., 2001). Stress‐coping theories emphasize an individual's voluntary attempts to cope with stress and the mastery of adaptive coping strategies and behaviors (e.g., Compas et al., 2001; Lazarus & Folkman, 1984). Meanwhile, psychophysiological perspectives highlight the function of the autonomic nervous system (ANS) as underlying an individual's physiological stress reactivity and denoting their capacity to engage in adaptive involuntary stress responses (e.g., Beauchaine, 2001; Porges, 2007). Multiple aspects of coping have been independently linked with psychopathology during adolescence (for meta‐analyses, see Compas et al., 2017; Lorber, 2004). Yet, relatively little attention has been paid to the integration of voluntary and involuntary stress responses, particularly with respect to specific kinds of stressors (e.g., interpersonal; Erath & Pettit, 2021). Therefore, the aims of the current study were to (a) examine the relationship between youths’ responses to peer and academic stress and their adjustment within these respective domains, and (b) focus on the joint implication of voluntary or purposeful coping efforts and involuntary or automatic physiological responses.

### 1.1 Linking coping with stress and youth adjustment

There are several related perspectives regarding the form and structure of coping, and the extent to which different forms of coping may be more or less effective in managing various challenging situations (Compas et al., 2001; Connor‐Smith et al., 2000; Skinner & Zimmer‐Gembeck, 2016a; Skinner et al., 2013). Common across these frameworks are the distinct families of coping (e.g., problem‐focused, emotion‐focused, avoidance, support‐seeking), as well as a higher order structure that groups related families of coping based on their function or purpose (Skinner et al., 2013), such as engagement–disengagement coping strategies (Compas et al., 2001; Connor‐Smith et al., 2000) or adaptive–maladaptive coping strategies (Skinner et al., 2013). The different terminology stems from the literature on different domains of stressors; engagement–disengagement is commonly used in the interpersonal stress domain (and their links with psychopathology; Compas et al., 2001, 2017) and adaptive–maladaptive is used in the academic challenges domain (stemming from a model of motivational resilience toward overcoming difficulties and setbacks; Skinner et al., 2013).

Despite some differences in terminology, there are many similarities across the families of coping within these higher order groups in the peer and academic coping literatures. For consistency and clarity in our paper, we use engagement coping (including both engagement and adaptive coping across the interpersonal and academic literatures, respectively) and disengagement coping (including disengagement and maladaptive coping across the interpersonal and academic literatures, respectively). Engagement coping refers to responses directed toward the stressor or one's reactions to the stressor that involve constructive, cognitive, and behavioral (re)engagement with the challenge, as well as strategies that regulate emotions, such as ameliorating negative emotions and promoting positive emotions toward the challenge (Compas et al., 2001; Skinner et al., 2013). In the peer domain, engagement coping strategies include problem‐solving, emotion regulation, emotional expression, positive thinking or cognitive restructuring, and acceptance (Compas et al., 2001; Connor‐Smith et al., 2000). Similarly, in the academic domain, such strategies include strategizing, help‐ or comfort‐seeking, self‐encouragement, and commitment (Skinner et al., 2013). In contrast, disengagement coping strategies reflect responses oriented away from the stressor or one's reactions to the stressor and that disengage an individual from dealing with the challenge (Compas et al., 2001; Skinner et al., 2013). In the peer domain, disengagement coping includes avoidance, denial, wishful thinking, and distraction (Compas et al., 2001; Connor‐Smith et al., 2000). Relatedly, in the academic domain, such strategies include escape, concealment, confusion, projection, rumination, and self‐pity (Skinner et al., 2013).

In addition to the conceptual overlap, similar patterns of associations have been found in a handful of studies linking youth coping and adjustment within each domain, such that engagement and disengagement coping are linked with better and worse adjustment, respectively. For example, greater self‐reported problem‐solving with peer stress (e.g., problematic relations) was linked concurrently with fewer teacher‐reported social problems (e.g., not liked, not getting along with others) for boys, as well as higher sociometric ratings of preference among less‐victimized youth (Kochenderfer‐Ladd & Skinner, 2002). In contrast, more distancing or avoidant coping in response to peer victimization was concurrently associated with more social problems (Kochenderfer‐Ladd & Skinner, 2002), as well as prospectively with decreased peer‐rated prosocial behaviors among girls and more peer‐rated victimization among initially victimized girls (Visconti & Troop‐Gordon, 2010). Similar to the peer domain, higher proportions of engagement coping strategies with academic challenges (e.g., problem‐solving, support‐seeking, positive rationalization) out of total strategies reported have been linked with better concurrent and prospective academic adjustment (e.g., less school burnout, higher academic competence, greater school engagement; Luo et al., 2016; Skinner et al., 2013, 2016). Conversely, disengagement coping strategies (e.g., escape, venting, fantasy, or wishful thinking) have been concurrently associated with poorer academic performance and higher burnout (Arsenio & Loria, 2014; Luo et al., 2016).

Together, existing studies on domain‐specific links between coping and adjustment have revealed a largely consistent pattern connecting the use of engagement or disengagement coping strategies and better or worse adjustment, respectively, within both the peer and academic domains. Yet, it is worth noting that these associations do not always emerge (e.g., Erath & Tu, 2014), and effect sizes for associations between coping and social/academic adjustment, albeit robustly significant, tend to be small or moderate (for a meta‐analysis, see Clarke, 2006). Therefore, additional work is warranted that identifies other contributing factors, such as  involuntary physiological responses to stress, that may help us understand the complex association between coping and adjustment.

### 1.2 The moderating role of cardiac autonomic functioning

In addition to volitional coping efforts, research on stress‐coping highlights involuntary, automatic, and biologically based processes as another essential aspect of one's stress response system (Compas et al., 2001). From a psychophysiological perspective, the ANS regulates an individual's homeostatic function (Porges & Furman, 2011). ANS responses, both arousal and reactivity, are important peripheral markers of the critical neural pathways to social behaviors and denote an individual's capacity to manage external or internal demands (Porges & Furman, 2011).

The ANS is composed of two main branches—sympathetic and parasympathetic—that exert largely antagonistic influence on cardiovascular functions (e.g., changes in heart rate and blood flow or pressure). According to Polyvagal Theory (Porges, 2007), in the face of environmental challenges, greater sympathetic nervous system (SNS) activity mobilizes the body to engage in “fight or flight” responses. For cardiac sympathetic functioning, shortened preejection period (PEP; time in milliseconds between electrical stimulus initiating ventricular contraction and opening of the aortic valve) is often considered the best available marker of increased beta‐adrenergic sympathetic influence on the heart (Berntson et al., 1994; Cacioppo et al., 1994; Sherwood et al., 1990; additional detailed explanations can be found in Zisner & Beauchaine, 2016). Accordingly, changes in PEP in response to external challenges (i.e., PEP reactivity) indicate one's ability to adapt to external demands and are thought to underlie approach‐oriented behaviors of the motivational system (Beauchaine, 2001). In comparison, the parasympathetic nervous system (PNS) underlies vagal regulation of cardiovascular activities, promoting rest, repair, and relaxation of the body and conservation of the body's resources during nonstressful circumstances (Porges, 2007). Withdrawal of parasympathetic influence in response to external demands allows more room for sympathetic influence on the heart (Porges, 2007). Respiratory sinus arrhythmia (RSA) or the component of heart rate variability linked to respiration (inhibitory PNS efference during inhalation, PNS withdrawal during exhalation) is proposed to provide a pure index of the capacity of parasympathetic cardiac control (Cacioppo et al., 1994; Porges, 1995; additional detailed explanations can be found in Zisner & Beauchaine, 2016). Changes in RSA from the resting state to environmental stimuli (i.e., RSA reactivity) thereby represent one's ability to manage external demands (Beauchaine, 2001).

Given the multifold nature of stress responses, incorporating cardiac autonomic functioning offers new insights into the coping‐adjustment association, as it can reveal the degree to which autonomic and voluntary responses intersect (e.g., facilitate or counteract each other) in jointly contributing to adjustment (Erath & Pettit, 2021). For example, within the peer domain, Erath and Tu (2014) found that youths’ self‐reported coping with peer stressors (i.e., lab‐induced peer evaluation, real‐life victimization) and RSA reactivity to peer evaluation was interactively associated with teacher‐reported social competence, such that disengagement coping in combination with lower RSA withdrawal (maintaining instead of withdrawing PNS response during stress) was linked with the lowest level of social competence. Within the academic domain, youths’ greater RSA withdrawal to a cognitive failure task (i.e., impossible puzzle) attenuated the association between poor executive functioning (which supports the development of coping; Skinner & Zimmer‐Gembeck, 2016b) and adult‐rated academic impairment (McQuade et al., 2017). Relatedly, greater heart rate variability after a lab‐induced school‐related stressor has been directly related to better academic performance among adolescents (e.g., Scrimin et al., 2019). Although PEP has received less attention in the coping‐adjustment literature, early‐institutionalized youth (due to abandonment), compared with their never‐institutionalized counterparts, showed prolonged PEP (blunted SNS reactivity) to social rejection, which in turn was associated with greater peer problems (Tang et al., 2022). These findings support PEP as another important indicator of stress response system functioning and its implications for youth adjustment.

Most importantly, because PNS and SNS work together to regulate cardiac responses to stress, models bridging the two aspects have been proposed. The autonomic space model (Berntson et al., 2008) proposes that individual differences in the reciprocal relations between the two branches can be characterized by either SNS or PNS dominance, and the *relative contributions* of PNS to SNS are indexed as *cardiac autonomic balance* (CAB). With sympathetically driven CAB (SNS > PNS; SNS dominance) as a result of attenuated PNS or overactive SNS, the body may be positioned to engage in cardiovascular responses to environmental perturbations and inhibit the return to a resting state. In contrast, a parasympathetically driven CAB (PNS > SNS; PNS dominance) reflects powerful homeostatic control or allostatic regulation. Moreover, individual differences in the *coactivity* in both branches—calculated as the sum of SNS and PNS contributions—can be characterized by coactivation (strong responses in both) or coinhibition (minimal responses in both) and are indexed as *cardiac autonomic regulation* (CAR). Although greater CAR or PNS–SNS coactivation may reflect the flexible engagement of both branches in reacting and regulating responses to perturbations, smaller CAR or PNS–SNS coinhibition may indicate limited activity in both branches and overall, an under‐aroused or disengaged state. Accordingly, CAB and CAR are considered to be independent indices of the ANS (Berntson et al., 2008). For both CAB and CAR, baseline measures denote the capacity of the respective physiological functioning, and the difference from baseline to laboratory tasks (e.g., emotionally salient video clips, parent–youth conflict discussion, demanding cognitive task) indicates the degree of one's physiological reactivity to environmental perturbations.

Compared with indicators of a single branch (PEP for SNS, RSA for PNS), CAB and CAR are conceptually more comprehensive in capturing an individual's stress response system (re)activity and thereby might serve as better predictors than PEP and RSA alone. Indeed, CAB and CAR have been highlighted as potential markers of physical health (Berntson et al., 2008), as well as psychopathological well‐being among clinical samples of youth and young adults (Brush et al., 2019; Bylsma et al., 2015; Choi et al., 2021; Cohen et al., 2020; Stone et al., 2020). Specifically, lower CAB reactivity (SNS > PNS from baseline to stress tasks) was linked with posttraumatic stress for young adolescents with high trauma exposure (Cohen et al., 2020). Among studies of young adults, low resting CAB was significantly linked with current major depressive disorder status (Brush et al., 2019) and higher depressive symptoms (Stone et al., 2020). Overall, lower CAB baseline and reactivity to stress, reflecting SNS dominance, appear to be linked with psychopathology (for one exception, see Bylsma et al., 2015). Meanwhile, evidence linking CAR indices with psychopathology is lacking (null findings in aforementioned studies), although some work has revealed that the nature (i.e., stressful vs. rewarding) of the assessment context might make a difference in the implication of CAB and CAR. For instance, among emerging adults, lower CAB reactivity to a stressful context predicted increasing trajectories of depression (for males only), whereas higher CAR reactivity (PNS–SNS coactivation) to a rewarding context was linked with lower depression (Choi et al., 2021).

Despite the growing focus on CAB and CAR as more comprehensive markers of cardiac ANS, no known study has extended their implications beyond psychopathology to the context of *social and academic adjustment* among *typically developing* youth. Further, the stress‐coping literature and the social information processing framework of coping suggest that involuntary physiological responses may underlie how youth process and represent the stressor, thereby enabling or constraining the tendency to initiate specific coping strategies as well as the effectiveness of implementing specific coping strategies (Connor‐Smith et al., 2000; Erath & Pettit, 2021). Thus, attempts are needed to elucidate the potential *interactive associations* between CAB/CAR and coping in relation to youth adjustment outcomes.

### 1.3 The current study

The current study investigated two aims within the peer and academic domains with a sample of typically developing youth across the transition to middle school (pre‐ and post‐transition, 7 months apart). First, we examined the independent associations linking coping and cardiac autonomic functioning pre‐transition with youth adjustment post‐transition. At the behavioral level, and informed by the stress‐coping literature (e.g., Luo et al., 2016; Visconti & Troop‐Gordon, 2010), we hypothesized that higher proportions of engagement coping out of total coping strategies employed would be associated with better adjustment. Adopting a within‐domain approach, coping with peer stress was used when linking social adjustment, and coping with academic stress was used when linking academic adjustment. At the physiological level, we assessed four indicators of cardiac autonomic functioning—CAB and CAR baseline during a resting task, as well as CAB and CAR reactivity in response to two separate mother–youth conversation tasks during which they discussed a recent peer and an academic challenge. Informed by the autonomic space model (Berntson et al., 2008) and some empirical evidence (Brush et al., 2019; Choi et al., 2021; Cohen et al., 2020; Stone et al., 2020), we hypothesized that greater CAB baseline and reactivity (PNS > SNS), as well as greater CAR baseline and reactivity (PNS–SNS coactivation), would predict better adjustment. Cardiac autonomic reactivity to a parent–youth conversation task about a *peer problem* was used when predicting social adjustment, and cardiac autonomic reactivity to discussing an *academic problem* was used when predicting academic adjustment. Of note, we did not have differential hypotheses for baseline and reactivity measures.

The second aim was to examine the interactive association between coping and cardiac autonomic functioning in the prediction of youth adjustment 7 months later. Building on the first aim, we took a within‐domain approach. Across both domains, we expected that coping and cardiac autonomic functioning would work synergistically and amplify the association of the other, such that the positive association between a higher proportion of engagement coping and youth adjustment would be stronger among youth exhibiting greater CAB and CAR baseline and reactivity.

## 2 METHODS

### 2.1 Participants

Data for this study came from a longitudinal study that followed mother–youth dyads during the transition to middle school. At Time 1 (T1; spring of 5th grade), participants included 100 youth (*M*_age_ = 11.04 years, *SD* = 0.33; 53% boys) and their mothers (*M*_age_ = 41.21 years, *SD* = 6.25; 96% biological), as well as 78 elementary school teachers (one teacher for each youth; 78% reports obtained in total; 98% of mothers provided parental permission). At Time 2 (T2; fall of 6th grade), 89 youth (*M*_age_ = 11.65 years, *SD* = 0.34) and their mothers (*M*_age_ = 41.65 years, *SD* = 5.68) returned, and a total of 76 middle school teachers participated (85% reports obtained in total; 98% of mothers provided parental permission).

Youth in our sample were 57% European American, 13% Hispanic or Latino/a, 10% African American, 6% Asian, and 14% other (e.g., mixed race). Mothers were 69% European American, 10% Hispanic or Latina, 8% African American, 8% Asian, 1% Native American, and 3% other (e.g., mixed race). For mothers’ highest level of education, 75% of mothers had a bachelor's degree or higher and 25% had less than a bachelor's degree. About 76% of mothers reported being married, 9% were divorced, and 15% were either single, separated, or had a significant other (not married). Approximately 63% of families reported an annual income ≥$75,000; 19% reported an income of $50,000–$75,000; 14% reported an income of $25,000–$50,000; and 4% reported an income of ≤$25,000.

### 2.2 Procedure

The study was approved by the institutional review board at the authors’ institution. Only procedures pertinent to this study are described. Data collection took place at the university laboratory in a rural to small‐urban Midwestern county in the United States. Data were collected across two cohorts separated by 1 year (T1 = Spring 2017 and 2018, respectively). Two time points were collected for each cohort approximately 7.40 months apart (*SD* = 0.88). For recruitment, permission was obtained to distribute informational letters through local elementary schools for students to take home. Families were also recruited through flyers posted in the community. Eligibility criteria included youth in 5th grade with one female parent/guardian willing to participate and English proficiency for observations of mother–youth conversation tasks. Exclusion criteria included youth with learning or developmental disabilities and known heart abnormalities due to the nature of larger project aims. Interested families were screened for eligibility over the phone; eligible families were invited to participate in a lab visit on the university campus. At both waves, consent/assent forms were reviewed separately for each family member; written consent/assent was obtained. Mothers also provided permission to contact teachers (T1 = fifth‐grade teacher, T2 = sixth‐grade teacher) to complete surveys. At both waves, families and teachers were compensated immediately following their participation.

At T1, during the 2‐h lab visit, mothers and youth each completed separate checklists of social (Connor‐Smith et al., 2000) and academic (Duchesne et al., 2012; Skinner et al., 2013; Wenz‐Gross et al., 1997) challenges adapted or developed for the project. For each challenge that was checked, participants rated how often the experience occurred to the youth (1 = *once or twice* to 4 = *all the time*) and how stressful it was for themselves (i.e., youth and mother, 1 = *not at all* to 4 = *very much*). Next, trained female undergraduate research assistants measured youths’ height and weight, as well as placed electrodes on youth for the recording of physiological data. After a 5‐min acclimation period for youth to adjust to the electrodes and for research assistants to inspect the signal quality, youths’ physiological activity was collected continuously during a 3‐min baseline task (watching a nature slideshow) and two conversation tasks (5‐min each, one social and one academic; order counterbalanced) with their mothers while in a face‐to‐face, seated position.

For each conversation task, two to four challenges that were checked by both the mother and youth and reported by the youth as being more stressful and/or frequent were provided as topic options for the conversation. Families were told, “Work together to select one topic to discuss for five minutes and approach the conversation just as you normally would.” The protocol for the conversation tasks was adapted from established procedures (e.g., family interaction tasks, Parent–Youth Interaction Task; Melby & Conger, 2001) but focused on youth peer problems and academic challenges rather than their conflict with parents. For the peer problem conversation, youth stress ratings for the selected topics, on average, were moderate (*M* = 2.42, *SD* = 0.94; scale of 1 = *not at all* to 4 = *very much*), and the four most common topics discussed were as follows: being around kids who are rude (*n* = 36); having problems with a friend (*n* = 16); feeling pressured to do something (*n* = 11); and being left out or rejected (*n* = 10). For the academic challenge conversation, youth stress ratings for the selected topics, on average, were also moderate (*M* = 2.36, *SD* = 0.97; scale of 1 = *not at all* to 4 = *very much*), and the four most common topics discussed were as follows: being bored in class (*n* = 22); not understanding something in class (*n* = 18); not doing as well as you wanted on an assignment, homework, quiz, or test (*n* = 14); and having difficulty with time management (*n* = 14). Lastly, mothers and youth completed questionnaires online through Qualtrics on laboratory computers in separate rooms. At T2, mother–youth dyads were contacted to participate in the second wave of the study where they were asked to complete surveys in separate rooms during a laboratory visit. At T1 and T2, after the family's lab visit, teachers were invited to complete online surveys. Teachers completed the surveys 25–28 days (*SD*s = 20.10–21.51, respectively) after the family's lab visit.

### 2.3 Measures

#### 2.3.1 Coping with social stress (T1)

At T1, youth reported on coping with peer stress using the Response to Stress Questionnaire—Social Stress (Connor‐Smith et al., 2000). The Engagement Coping subscale was composed of six specific families of coping (three items per family), including problem‐solving, emotion regulation, emotional expression, positive thinking, cognitive restructuring, and acceptance (α = .85). The Disengagement Coping subscale was composed of four families of coping (three items per family), including avoidance, denial, wishful thinking, and distraction (α = .60). Items were rated on a 4‐point scale (1 = *not at all* to 4 = *a lot*; for a list of all items, refer to Connor‐Smith et al., 2000). Because youth likely employ a combination of strategies to cope with stress, allocation or proportion scores that represent the balance of engagement relative to overall coping (vs. the use of engagement coping alone) may better predict adjustment outcomes (Cheng et al., 2014). Thus, we computed the proportion of engagement coping (i.e., engagement coping divided by the sum of engagement and disengagement coping) as an indicator of youth coping with peer stress. Despite the lower reliability for the disengagement subscale alone, the sum of engagement and disengagement coping with peer stress showed satisfying reliability (α = .85).

#### 2.3.2 Coping with academic stress (T1)

At T1, youth reported on coping with academic failures using the Multidimensional Measure of Coping Questionnaire (Skinner et al., 2013). The Adaptive Coping subscale consisted of four families of coping (five items per family), including strategizing, help‐seeking, comfort‐seeking, and self‐encouragement (α = .91). The Maladaptive Coping subscale consisted of three families of coping, including escape (five items), concealment (two items), and rumination (five items) (α = .74). Items were rated on a 4‐point scale (1 = *not at all true* to 4 = *very true*; for items, refer to Skinner et al., 2013). Similar to coping with peer stress, we computed the proportion of adaptive coping (i.e., adaptive coping divided by the sum of adaptive and maladaptive coping) as an indicator of youth's coping with academic stress. For consistency of terminology, in this study, we substituted “adaptive” with “engagement” when referring to coping with academic stress. Overall, coping with academic stress showed strong reliability (α = .87).

#### 2.3.3 Cardiac autonomic balance and regulation (T1)

For physiological data acquisition, we used a Bioamp data acquisition system (MindWare Technologies, Inc., Gahanna, OH). Thoracic impedance data were collected using a four‐spot impedance configuration (Sherwood et al., 1990) in which electrodes were placed at the apex and base of the thorax, and dual electrodes were placed on the back that were 1½ inches above and below the thorax electrodes. Cardiac data were collected with a modified lead‐II configuration (Berntson et al., 1997) in which electrodes were placed on the right clavicle and left and right ribs. Specifically, Ag‐AgCl electrodes (1′ foam, 7% chloride gel) from MindWare Technologies, Inc. were used. Data were sampled at 1000 Hz.

PEP and RSA scores were quantified in 1‐min intervals from the impedance cardiography (or the cardiac impedance signal) and electrocardiogram (ECG) using IMP Analysis 3.1.6 and HRV Analysis 3.1.5, respectively (MindWare Technologies, Inc., Gahanna, OH). Trained research assistants visually inspected the waveforms to verify or edit R peaks (for PEP and RSA) and B points (for PEP). Specifically, misidentified R peaks and B points were manually corrected based on guidelines in the technical manuals and training provided by MindWare Technologies (https://mindwaretech.com/). PEP was calculated as the time interval (units = milliseconds) between the Q‐point of the ECG and the B‐point of the *dZ*/*dt* signal. For each participant, one out of two B‐point calculation methods (i.e., Max Slope Change with a block size of 1; Percent *dZ*/*dt* Time [peak = 55] + C [4]) was used consistently across all tasks within a participant depending on the morphology of *dZ*/*dt* signal (i.e., the presence of a visible B notch) in the ensemble average. RSA scores [units = natural logarithm of milliseconds squared or ln(ms)^2^] were quantified using the spectral analysis method (Berntson et al., 1997). Respiration was derived using the cardiac impedance signal *Z*_0_. The high‐frequency/RSA band (Hz) used to analyze the data was 0.24−1.04 per recommendations for age‐appropriate respiration frequencies (Shader et al., 2018). The few cases of artifacts were manually corrected following standard procedures (Berntson et al., 1997).

For each task (i.e., baseline, conversations), PEP and RSA scores were averaged across all intervals. PEP and RSA reactivity scores were computed as the difference between the conversation and baseline scores. Following established procedures (Berntson et al., 2008), baseline and reactivity scores were standardized; CAB was computed as RSAz minus (–PEPz), such that higher values reflect greater PNS to SNS functioning, and lower values reflect greater SNS to PNS functioning. CAR was computed as RSAz plus (–PEPz), such that higher values reflect greater coactivation, and lower values reflect less coactivation or more coinhibition.

#### 2.3.4 Social adjustment (T1 and T2)

Adopting a comprehensive approach to capturing youth adjustment in the social domain, we assessed, from multiple informants, six related but distinct aspects of youth social adjustment—friendship quality, peer acceptance, social skills, social competence, social status, and peer victimization. Youth completed the Friendship Quality Questionnaire (Parker & Asher, 1993; 25 items; e.g., “We make each other feel important and special”) and rated how true each item was about their closest friends (0 = *not at all true* to 4 = *really true*) (α_T1/T2_ = .95/.95). Mothers reported on youths’ peer acceptance using the Checklist of Peer Relations (Dodge, 1986; Dodge & Coie, 1987; six items; e.g., “Other children like my child and seek him or her out”) on a 5‐point scale (0 = *never true* to 4 = *almost always true*) (α_T1/T2_ = .83/.84). Mothers also rated youths’ social skills during interpersonal situations (Coie & Dodge, 1988; seven items; e.g., “Being socially aware of what is happening in a situation”) on a 5‐point scale (1 = *very poor* to 5 = *very good*) (α_T1/T2_ = .87/.89). Further, teachers completed the 25‐item Teacher Ratings of Child Behavior Change (Conduct Problems Prevention Research Group [CPPRG], 1990) which is designed to assess youth's general social competence such as emotional understanding and communication, self‐control, friendship building, and social problem‐solving. Items were rated on a 5‐point scale (0 = *not at all* to 4 = *very well*) (α_T1/T2_ = .98/.97). Teachers also reported on youths’ peer victimization (physical, relational, and verbal) using the Social Experiences Questionnaire (Crick & Grotpeter, 1996; Cullerton‐Sen & Crick, 2005; 11 items, e.g., “This child is the target of rumors or gossip in their peer group”) on a 5‐point scale (1 = *never* to 5 = *almost always*) (α_T1/T2_ = .88/.92). To align peer victimization with the direction of other aspects of social adjustment, all items were reverse coded before computing the overall composite where higher scores indicated lower peer victimization. Finally, teachers reported on youths’ social status (i.e., unpopular, neglected, rejected; Rudolph & Clark, 2001) on a 7‐point scale (1 = *not at all* to 7 = *extremely*) (α_T1/T2_ = .84/.77). These items were also reverse coded before computing the overall composite such that higher scores indicated higher social status.

In general, six aspects of social adjustment were positively correlated with each other at each time point and were stable across time points (see Table S1A). Thus, for each youth and at each time point, one overall social adjustment composite was created by standardizing the scores for each measure and then averaging across measures (see notes under Table S1A for results from confirmatory factor analyses that further support the use of a single composite for social adjustment at each time point).

#### 2.3.5 Academic adjustment (T1 and T2)

We assessed youths’ academic subject performance, school and academic adjustment, school engagement, and academic functioning as indicators of their overall academic adjustment from multiple informants. Youth rated how well they perform in English/language, reading, mathematics, science, and social science/history using the Mock Report Card (Pierce et al., 1999) on a 5‐point scale (1 = *failing* to 5 = *excellent*) (α_T1/T2_ = .72/.77). Mothers reported on the School Adjustment Scale—Parent (Revised) (CPPRG, 2003; nine items) to indicate youth's school adjustment (e.g., “My child had a good year at school”) and academic adjustment (e.g., “My child had an easy time handling the new academic demands made on them”) on a 5‐point scale (1 = *strongly disagree* to 5 = *strongly agree*) (α_T1/T2_ = .84/.79). Teachers completed three items in the Research Assessment Package for Schools (Institute for Research & Reform in Education, 1998; e.g., “In my class, this student seems tuned in”) to indicate youths’ school engagement on a 4‐point scale (1 = *not at all true* to 5 = *very true*) (α_T1/T2_ = .85/.70). Teachers also completed the Academic Functioning subscale in the Social Behavior Rating Scale (Schwartz et al., 2002; three items; e.g., “This child has difficulties with school work”) on a 5‐point scale (1 = *almost never true* to 5 = *almost always true*) (α_T1/T2_ = .86/.81).

All four aspects of academic adjustment were positively correlated with each other at each time point and were stable across time points (see Table S1A). Therefore, for each youth and at each time point, one overall academic adjustment score was created by standardizing the scores for each measure and then averaging across measures (see notes under Table S1A for results from confirmatory factor analyses that further support the use of a single composite for academic adjustment at each time point).

#### 2.3.6 Demographic covariates (T1)

Mother reports of youth age (calculated with the date of birth and the date of lab visit), gender, and race/ethnicity were included as covariates given existing evidence documenting potential differences in social and academic adjustment related to these demographic variables (e.g., Akos & Galassi, 2004; Brass et al., 2019; Graham, 2018; Pomerantz et al., 2002). Study cohort was also included as a covariate. Effect coding (Aiken & West, 1991) was adopted for handling dichotomous or categorical demographic variables based on recommended practices to avoid selecting a reference group (or centering one group) toward being more inclusive in the interpretation of predictors’ coefficients (Mayhew & Simonoff, 2015). Accordingly, gender was coded as girl = −1 and boy = 1; cohort was coded as cohort 1 = −1, cohort 2 = 1. For race/ethnicity, categories were separated into four “contrast variables” with each of the four selected groups: African American, Asian, European American, and Hispanic/Latino/a. These four groups were contrasted with “Other” (e.g., mixed race). For each contrast variable, the selected group was coded as 1, the “other” group as −1, and the remaining three groups as 0.

### 2.4 Data analytic plan

Preliminary analyses were conducted to examine study variable distributions, descriptive statistics, and correlations. To examine the independent and interactive roles of coping and cardiac autonomic functioning in youth subsequent adjustment, separate stepwise multiple regression models were fitted for each indicator of cardiac autonomic functioning (CAB and CAR at baseline and reactivity) and for each domain (social and academic), resulting in eight models in total at the final step. For each model, demographic covariates (i.e., youth age, gender, race/ethnicity), study cohort, and T1 levels of adjustment in the respective domain were entered first, followed by the main effect of T1 coping and cardiac autonomic functioning as separate steps. The interaction between them was entered as the last step. All continuous predictors were mean centered before creating interaction terms and included in the models. To probe significant interaction effects, simple slopes for youth coping were tested and plotted at ±1 *SD* of the mean on cardiac autonomic functioning (Aiken & West, 1991; Preacher et al., 2006). Regions of significance were also obtained to further determine (a) levels of coping at which the association between cardiac autonomic functioning and adjustment became significantly different and (b) levels of cardiac autonomic functioning at which the association between coping and adjustment became significantly different (Preacher et al., 2006).

## 3 RESULTS

### 3.1 Preliminary analyses

Data were checked (outlier, skewness, missingness) and preliminary analyses (descriptive statistics, bivariate correlations, and *t*‐tests) were conducted in IBM SPSS Statistics (Version 28.0). For missingness on key study variables, one youth did not report on their coping with academic challenges at T1. Missing physiological data were due to bad signals during the baseline task (*n* = 5) or failure to record due to technical difficulties (*n* = 1). Attrition resulted in missing data (*n* = 11) on social and academic adjustment at T2. No significant differences in key study variables were found for youth with and without physiological data, as well as for those who returned and did not return at T2. Overall, Little's Missing Completely At Random (MCAR) test among all variables included in the model was nonsignificant, *χ* ^2^ (57) = 54.11, *p* = .584, indicating that the missing data mechanism was likely MCAR.

As shown in Table 1, social and academic adjustment outcomes were positively correlated across domains and time points. Older youth reported better adjustment in general, except for T1 academic adjustment. Engagement coping with social and academic stress was positively correlated, and higher proportions of engagement coping in both domains were associated with better social and academic adjustment at T1 and T2. Interestingly, higher proportions of engagement coping with academic stress were associated with lower CAB baseline (SNS > PNS). Independent sample *t*‐tests revealed no difference in continuous study variables by study cohort. However, girls reported higher proportions of engagement coping with academic stress, lower CAB baseline, and better social adjustment at T1 and T2 (see Table 2 for a detailed summary of gender group differences). Further, results from one‐way ANOVAs indicated no overall difference in key study variables by race/ethnicity groups.

**TABLE 1:** Descriptive statistics and bivariate correlations among key study variables

		1	2	3	4	5	6	7	8	9
1	T1 age	–								
2	T1 % engagement coping—peer	.08	–							
3	T1 % engagement coping—academic	.16	.40***	–						
4	T1 CAB baseline	−.02	−.18	−.20*	–					
5	T1 CAR baseline	.05	.05	−.01	−.02	–				
6	T1 social adjustment	.23*	.28**	.25*	.02	.00	–			
7	T1 academic adjustment	.10	.29**	.26*	−.07	−.03	.55***	–		
8	T2 social adjustment	.30**	.34***	.32**	−.03	−.02	.78***	.47***	–	
9	T2 academic adjustment	.27*	.35***	.24*	−.02	−.04	.56***	.64***	.70***	–
*M*		11.04	0.55	0.60	0.03	0.03	−0.04	−0.02	0.00	0.00
*SD*		0.33	0.04	0.07	1.48	1.31	0.76	0.78	0.67	0.80
Minimum		10.08	0.44	0.42	−4.17	−3.31	−2.87	−2.18	−2.35	−2.95
Maximum		12.17	0.66	0.75	3.81	2.79	1.01	1.15	1.23	1.55
*N*		100	100	99	94	94	100	100	89	89

*Note*: T1% engagement coping represented the proportion of engagement coping (i.e., engagement coping divided by the sum of engagement and disengagement coping) at T1. Youth CAB/CAR and adjustment outcomes were created using standardized variables (*z*‐scores).

Abbreviations: CAB, cardiac autonomic balance (higher scores = PNS > SNS); CAR, cardiac autonomic regulation (higher scores = coactivation); T1/T2, Time 1/Time 2.

*p < .05; **p < .01; ***p < .001.

**TABLE 2:** Descriptive statistics and tests of key study variables by youth gender

		*M* (*SD*)		Independent samples *t*‐tests			
		**Girl** (*n* = 47)	**Boy** (*n* = 53)	*t*	*df*	*p*	95% CI
1	T1 age	11.10 (0.27)	10.99 (0.37)	1.66	98	.100	[–0.02, 0.24]
2	T1 % engagement coping—peer	0.55 (0.04)	0.54 (0.05)	1.19	98	.237	[–0.01, 0.03]
3	T1 % engagement coping—academic	0.62 (0.06)	0.58 (0.07)	2.98	97	.004	[0.01, 0.07]
4	T1 CAB baseline	−0.33(1.53)	0.32 (1.39)	−2.16	92	.034	[−1.25, –0.05]
5	T1 CAR baseline	−0.22 (1.30)	0.23 (1.30)	−1.67	92	.098	[–0.99, 0.08]
6	T1 social adjustment	0.14 (0.61)	−0.20 (0.84)	2.34	98	.021	[0.05, 0.64]
7	T1 academic adjustment	0.06 (0.65)	−0.09 (0.87)	0.96	98	.339	[–0.16, 0.46]
8	T2 social adjustment	0.24 (0.51)	−0.23 (0.72)	3.50	87	.001	[0.20, 0.73]
9	T2 academic adjustment	0.07 (0.71)	−0.07 (0.87)	0.85	87	.400	[–0.19, 0.48]

*Note*: T1 % engagement coping represented the proportion of engagement coping (i.e., engagement coping divided by the sum of engagement and disengagement coping) at T1. Youth CAB/CAR and adjustment outcomes were created using standardized variables (*z*‐scores).

Abbreviations: CAB, cardiac autonomic balance (higher scores = PNS > SNS); CAR, cardiac autonomic regulation (higher scores = coactivation); T1/T2, Time 1/Time 2.

Given that most findings from the main model tests emerged for baseline but not reactivity measures, we focused on presenting and interpreting baseline findings below. For CAB and CAR reactivity, descriptive statistics and bivariate correlations are presented and described in the Supporting Information (see Table S2A), and related main model test results are summarized in the Supporting Information as well (see Table S3A).

### 3.2 Main model tests

Tests of our main model were conducted in IBM SPSS Amos (Version 28.0). Missing data were handled using full information maximum likelihood estimation (Acock, 2005). Results for models with baseline measures are presented in Table 3.

**TABLE 3:** Regressions for coping and cardiac autonomic baseline predicting youth adjustment

	T2 social adjustment					T2 academic adjustment				
Effects for T1 predictors	*β*	*B*	*SE*	*p*	*R* ^2^/Δ*R* ^2^	*β*	*B*	*SE*	*p*	*R* ^2^/Δ*R* ^2^
***Covariates***					63.6%					53.3%
Age	0.14	0.26	0.12	.034		0.19	0.48	0.18	.009	
Gender	−0.17	−0.11	0.04	.008		−0.01	0.00	0.06	.944	
African American	0.03	0.04	0.08	.608		−0.05	−0.09	0.12	.484	
Asian	−0.04	−0.06	0.09	.500		−0.07	−0.14	0.14	.310	
European American	−0.10	−0.09	0.06	.128		−0.03	−0.04	0.08	.661	
Hispanic or Latino	−0.06	−0.07	0.08	.348		0.03	0.04	0.12	.709	
Study cohort	−0.09	−0.06	0.04	.147		−0.20	−0.17	0.06	.006	
Adjustment	0.75	0.63	0.05	<.001		0.67	0.71	0.08	<.001	
*Main predictor*										
Coping (% engagement)	0.15	2.22	0.93	.017	0.4%	0.12	1.42	0.88	.105	0.4%
*Moderator (separate models)*										
CAB baseline	0.003	0.001	0.03	.960	0%	0.03	0.02	0.04	.684	0.2%
CAR baseline	−0.02	−0.01	0.03	.795	0%	−0.05	−0.03	0.05	.466	0.3%
*Interaction (separate models)*										
Coping × CAB baseline	−0.13	−1.44	0.69	.037	1.4%	0.02	0.15	0.56	.791	−0.1%
Coping × CAR baseline	0.16	1.90	0.76	.013	2.8%	0.16	1.38	0.63	.028	0.7%

*Note*: Coping (% engagement) represented the engagement coping divided by the sum of engagement and disengagement coping. Coping with peer and academic stress was entered in models predicting social and academic adjustment, respectively. Coefficients reported from step of entry.

Abbreviations: CAB, cardiac autonomic balance (higher scores = PNS > SNS); CAR, cardiac autonomic regulation (higher scores = coactivation); T1/T2, Time 1/Time 2.

#### 3.2.1 Coping with peer stress and CAB/CAR baseline predicting social adjustment

As shown in Table 3, across the two social adjustment models (for CAB and CAR), older youth and girls had better social adjustment at T2, but T2 social adjustment did not significantly differ by race/ethnic groups or study cohort. Consistent with our hypotheses, a higher proportion of T1 engagement coping with peer stress was associated with better T2 social adjustment above and beyond the association with T1 social adjustment. Although main effects for the baseline CAB/CAR measures did not emerge, CAB and CAR baseline moderated the association between T1 engagement coping and T2 social adjustment, explaining 1.4% and 2.8% of the unique variance, respectively.

**FIGURE 1:**
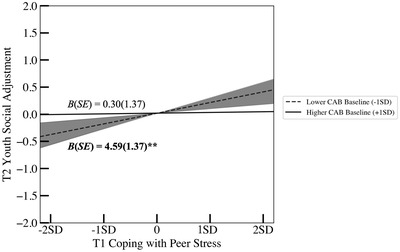
Associations between coping with peer stress and social adjustment as a function of cardiac autonomic balance (CAB) baseline. Associations reported controlled for covariates. Social adjustment indicators include friendship quality, peer acceptance, social skill, social competence, social status, and peer victimization. The dark gray‐shaded area (63.8% of the sample youth) around lower CAB baseline (−1 *SD*) refers to the region of significance analysis with respect to the moderator (i.e., CAB baseline).

For the moderating effect of CAB baseline, follow‐up simple slope tests revealed that a higher proportion of engagement coping with peer stress at T1 was associated with better social adjustment at T2 for youth who showed *lower* levels of CAB baseline (SNS > PNS), *B* (*SE*) = 4.59 (1.37), *p* = .001, but not for those who showed higher levels of CAB baseline (PNS > SNS), *B* (*SE*) = 0.30 (1.37), *p* = .826 (Figure 1). Further, regions of significance tests with respect to the moderator revealed that the lower bound of significance for CAB baseline was 0.26 *SD* above the mean, indicating that the positive association between T1 engagement coping and T2 social adjustment became significant when the level of CAB baseline was lower than 0.26 *SD* above the mean (63.8% of the sample youth; dark gray‐shaded area in Figure 1). Of note, the upper bound of this region of significance analysis (i.e., 23.91 *SD*s above the mean) was far beyond the highest observed value of CAB baseline in our sample. The lower and upper bounds of the regions of significance analysis with respect to the predictor (i.e., coping) were also slightly outside the observed value of T1 coping with peer stress in sample data (2.67 and 2.89 *SD*s below and above its mean, respectively) and thus were not presented in Figure 1.

**FIGURE 2:**
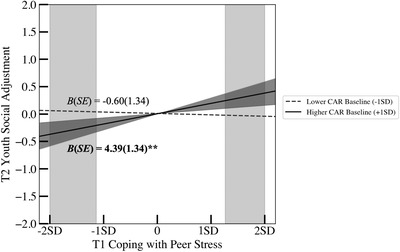
Associations between coping with peer stress and social adjustment as a function of cardiac autonomic regulation (CAR) baseline. Associations reported controlled for covariates. Social adjustment indicators include friendship quality, peer acceptance, social skill, social competence, social status, and peer victimization. The dark gray‐shaded area (46.8% of the sample youth) around higher CAR baseline (+1 *SD*) refers to the region of significance analysis with respect to the moderator (i.e., CAR baseline). The light gray‐shaded areas (12.0% and 11.0% of the sample youth on the left and right side, respectively) represent the regions of significance analysis with respect to the predictor (i.e., coping).

For the moderating effect of CAR baseline, follow‐up simple slope analyses revealed that a higher proportion of engagement coping with peer stress at T1 was associated with better social adjustment at T2 at *higher* levels of CAR baseline (coactivation), *B* (*SE*) = 4.39 (1.34), *p* = .002, but nonsignificant at lower levels of CAR baseline (coinhibition), *B* (*SE*) = −0.60 (1.34), *p* = .658. Further, regions of significance tests with respect to the moderator revealed that the upper bound of significance for CAR baseline was 0.05 *SD* below the mean, such that the positive association between T1 engagement coping and T2 social adjustment became significant when the level of CAR baseline was greater than 0.05 *SD* below the mean (46.8% of the sample youth; dark gray‐shaded area in Figure 2). The lower bound of this regions of significance analysis (i.e., 4.11 *SD*s below the mean) was beyond the lowest observed value of CAB baseline in our sample and thus was not presented in Figure 2. Additionally, regions of significance tests with respect to the main predictor revealed that the lower and upper bounds of significance for engagement coping with social stress were 1.13 *SD*s below the mean and 1.26 *SD*s above the mean, respectively. Thus, in our sample, the negative association between T1 CAR baseline and T2 social adjustment became significant when T1 engagement coping was lower than 1.13 *SD*s below the mean (12.0% of the sample youth; left side, light gray‐shaded area in Figure 2) or greater than 1.26 *SD*s above the mean (11.0% of the sample youth; right side, light gray‐shaded area in Figure 2).

#### 3.2.2 Coping with academic stress and CAB/CAR baseline predicting academic adjustment

As shown in Table 3, across the two academic adjustment models, older youth and youth from cohort 2 had better T2 academic adjustment, whereas no difference was found by gender or race/ethnicity groups. As expected, academic adjustment at T1 and T2 was positively associated. Surprisingly, T1 engagement coping with academic stress, albeit trending, was not significantly associated with T2 academic adjustment (*p*s < .10). Main and moderation effects of CAB baseline did not emerge. Although a main effect of CAR baseline did not emerge, CAR baseline moderated the association between T1 engagement coping and T2 academic adjustment, explaining 0.7% of the unique variance.

**FIGURE 3:**
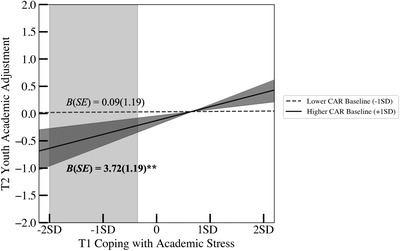
Associations between coping with peer stress and social adjustment as a function of cardiac autonomic balance (CAB) baseline. Associations reported controlled for covariates. Academic adjustment indicators include subject performance, school and academic adjustment, school engagement, and academic functioning. The dark gray‐shaded area (53.2% of the sample youth) around higher CAR baseline (+1 *SD*) refers to the region of significance analysis with respect to the moderator (i.e., CAR baseline). The light gray‐shaded area (32% of the sample youth) represents the regions of significance analysis with respect to the predictor (i.e., coping).

Follow‐up simple slope tests for the moderating effect of CAR baseline revealed that a higher proportion of T1 engagement coping with academic stress was associated with better T2 academic adjustment at *higher* levels of CAR baseline (coactivation), *B* (*SE*) = 3.72 (1.19), *p* = .002, but not at lower levels of CAR baseline (coinhibition), *B* (*SE*) = 0.09 (1.19), *p* = .939 (Figure 3). Further, regions of significance tests revealed that the upper bound of significance for CAR baseline was 0.11 *SD* below the mean, such that the positive association between T1 engagement coping and T2 academic adjustment became significant when CAR baseline was greater than 0.11 *SD* below the mean (53.2% of the sample youth; dark gray‐shaded area in Figure 3). The lower bound of this region of significance analysis (i.e., 11.49 *SD*s below the mean) was far beyond the lowest observed value of CAB baseline in our sample and thus was not depicted in Figure 3. Additionally, regions of significance tests with respect to the main predictor revealed that the lower bound of significance for engagement coping was 0.35 *SD* below the mean, indicating that the negative association between T1 CAR baseline and T2 youth academic adjustment became significant when T1 engagement coping with academic stress was lower than 0.35 *SD* below the mean (32.0% of the sample youth; light gray‐shaded are in Figure 3). The upper bound of this region of significance analysis (i.e., 7.52 *SD*s above the mean) was beyond the highest observed value of coping with academic stress in our sample and thus was not depicted in Figure 3.

## 4 DISCUSSION

Recent studies have demonstrated the need to investigate how involuntary physiological functioning may interact with the use of voluntary coping strategies to predict youths’ social and academic adjustment (e.g., Erath & Tu, 2014; McQuade et al., 2017). Moreover, CAB and CAR, compared to indicators of a single branch of the ANS, have been increasingly emphasized as more comprehensive cardiac markers of psychopathology among clinical samples of youth (e.g., Bylsma et al., 2015; Cohen et al., 2020). Yet, the implications of CAB and CAR among typically developing, community populations in the context of coping with daily stressors have received much less attention. Therefore, enriching the stress‐coping literature from a psychophysiological perspective, we examined the independent and interactive associations between youth coping and cardiac autonomic functioning in the prediction of adjustment outcomes, within the peer and academic domains during the transition to middle school. Consistent with hypotheses and prior literature (e.g., Compas et al., 2017; Skinner et al., 2013), the proportion of engagement coping in the peer domain was positively linked with better social adjustment after the middle school transition. The overall coping–adjustment association in the academic domain, although in the same direction, was nonsignificant. Findings also supported our hypothesis that the prospective association between coping and adjustment was qualified by youth cardiac autonomic functioning, although only CAR baseline consistently emerged as a moderator for both domains. As expected, higher proportions of engagement coping predicted better adjustment among youth who exhibited *higher* but not lower CAR baseline. Surprisingly, the same positive association linking coping with peer stress and social adjustment was found among youth who showed *lower* but not higher CAB baseline. Finally, CAB or CAR reactivity to mother–youth conversations did not moderate the coping–adjustment association in either domain.

Although direct associations linking cardiac autonomic baseline and reactivity with youth social and academic adjustment did not emerge, partially consistent with hypotheses, CAB and CAR baseline emerged as moderators of the youth coping–adjustment links. As expected, among youth who showed higher CAR baseline (greater coactivation capacity), higher proportions of engagement coping with peer and academic challenges were prospectively associated with better social and academic adjustment, respectively. In contrast, no association was found in either domain among youth who showed lower CAR baseline (limited coactivation capacity or tendency to coinhibit). Although PNS–SNS coactivation measured at baseline reflects the greater capacity to mobilize bodily resources in response to potential challenges, limited PNS–SNS coactivation may indicate under‐arousal and less capacity to engage with or respond to environmental perturbations (Berntson et al., 2008). Indeed, one machine learning study identified a cluster of ANS responses among adults (including PEP and RSA among multiple indices) characterized by a challenge‐like pattern of physiological reactivity—reflective of greater PNS–SNS coactivation during a cognitive performance task (i.e., mental arithmetic in the presence of an evaluator) (Wormwood et al., 2019). This ANS pattern was proposed to reflect how the body is mobilized but also under strong control (Wormwood et al., 2019), thereby potentially maximizing the effectiveness of engagement coping. Another cluster of ANS responses emerged that was characterized by a threat‐like pattern of physiological reactivity (more modest PNS–SNS coactivation), putatively signaling the inability to handle the demand (Wormwood et al., 2019), which might constrain the benefits of engagement coping strategies for youth adjustment.

A closer examination of these interactions suggests that a combination of *more engagement* coping and *higher CAR* baseline may reflect matched voluntary and involuntary coping responses in preparation toward responding to challenges. We found that such a combination was linked with the best adjustment. These findings demonstrate the potential importance of coordination among multiple facets of the stress response system whereby physiological functioning that supports youths’ capacity to actively approach challenges appears to be complementary to youths’ voluntary engagement coping strategies, such that both are coordinated toward the same “goal” of attending to the peer stressor. Conversely, a combination of higher CAR baseline and *less engagement* coping may undermine youth's social and academic adjustment because of a mismatch between the bodily state characterized by high approach tendencies and behavioral strategies that position youth toward more avoidance or less approach. To our knowledge, this is the first study to document evidence about CAR on social and academic adjustment among typically developing youth, and our findings suggest that the variability in cardiac autonomic regulation capacity may help to maximize or attenuate the effectiveness of voluntary coping with daily stressors rather than directly predicting outcomes themselves. Findings further demonstrate the need to integrate physiological and behavioral responses to daily stresorses, as the two are intertwined in contributing to youth adjustment during critical developmental transitions.

For CAB baseline, a different pattern emerged. CAB baseline is thought to reflect the reciprocal or relative influence between PNS and SNS, with greater CAB reflecting PNS dominance and better self‐regulatory abilities and lower CAB reflecting SNS dominance and a threat‐like bodily state, preparing individuals to engage in stress responses (e.g., Berntson et al., 2008; Wormwood et al., 2019). Existing work among clinical samples of youth has identified lower CAB as a risk factor for psychopathological outcomes (Brush et al., 2019; Choi et al., 2021; Cohen et al., 2020; Stone et al., 2020), and SNS dominance at baseline as an indicator of diminished PNS activity and poorer self‐regulatory capacity (Beauchaine, 2001). However, we did not find a direct link between CAB baseline and social/academic adjustment among our community sample of youth. Moreover, and contrary to our hypothesis, more engagement coping with peer stress was associated with better social adjustment emerged when youth showed *lower* compared with higher CAB baseline. To explain, greater as compared to lower proportions of engagement coping efforts (e.g., support‐seeking, cognitive reconstructing) may compensate or offset such autonomic tendencies and help contribute to better social adjustment. It is also possible that given the lab‐based nature of this study, cardiac SNS dominance during baseline may reflect an overall anticipatory or preparation state ahead of the peer and academic challenge discussions following the baseline period, and in coordination with higher proportions of engagement coping, could also reflect some degree of compatibility toward better social adjustment. Nonetheless, we caution against overinterpreting this result given that, in our sample, differences in the level of CAB baseline did not seem to indicate significant variations in social adjustment regardless of youth's use of engagement coping.

Moreover, despite our novel, contextualized assessments of physiological *reactivity* (i.e., mother–youth discussion of peer and academic challenges) in addition to baseline measures, we did not find main or moderation effects for CAB or CAR reactivity. One explanation could be that our conversation task, albeit pertinent to youths’ real‐life stressors, is not well‐suited for consistently inducing stress specifically and/or might not reflect youths’ physiological responses during actual stressful encounters in their daily lives. Prior studies measuring CAB and CAR reactivity employed psychological (e.g., sad film), physical (e.g., handgrip), or interpersonal (e.g., parent–child conflict discussion) tasks that are stressful, or at least challenging, for youth in nature (e.g., Bylsma et al., 2015; Cohen et al., 2020). In comparison, although our conversation tasks were potentially more representative of naturally occurring parent–youth interactions, the wide range of interaction dynamics displayed (e.g., recollecting details of experience, analyzing the situation, generating solutions, having disagreements) make it more complex than direct stress‐eliciting tasks, and thus might differentially relate to subsequent adjustment outcomes. Nevertheless, null findings may inform future research on social and academic adjustment, elucidating that the interpretation of physiological indicators may be highly dependent and sensitive to context.

One major strength of our study is the within‐domain approach focusing on youths’ peer and academic lives: (a) assessments of coping specifically targeted challenges in each domain (vs. general coping styles); (b) assessments of adjustment were comprehensive, from multiple informants, and reflected the complex composition of outcomes with respect to each domain; (c) two‐wave, short‐term longitudinal design allowed examination of these within‐domain processes across the critical transition to middle school. Still, it is worth noting that although the pattern of findings appeared to be similar overall across both domains, as discussed above, our findings revealed that the association with coping was weaker for academic as compared to social adjustment. We would like to highlight that the conceptualization and respective measurements for coping and adjustment outcomes are parallel, yet distinct across domains, which may help contextualize this weaker association. For example, within the academic domain, coping alone might have limited influence on subject performance, whereas other factors such as motivation, self‐efficacy, interest, and preexisting cognitive abilities (e.g., reading/language, planning) may also play significant roles (e.g., Skinner et al., 2013).

Our study has several limitations, and relatedly, we highlight a few important areas for future research. First, our community sample of middle‐class, highly educated families limits the generalizability of findings to more socioeconomically disadvantaged populations. Second, our sample size is inevitably modest due to the nature of longitudinal data collection involving parent–youth observations and physiological activities. Although the racial/ethnic background of our sample is relatively diverse and representative of the area where the study was conducted, we are underpowered to further examine *within‐group* variations in the use of coping strategies and their associations with adjustment, despite the increasing emphasis on understanding culturally congruent coping strategies and their unique effects in shaping adolescent adjustment within a certain minority group (e.g., McDermott et al., 2019). Future studies applying a psychophysiological perspective to stress‐coping research are needed to examine these processes within larger minority subgroups. Third, we assessed the general use of coping strategies across a range of challenges within each domain, but recent studies highlight the *flexibility* in coping across different stressors (e.g., selecting appropriate strategies depending on different or changing contextual demands) as uniquely predictive of individual's psychological adjustment (Cheng et al., 2014; Zimmer‐Gembeck et al., 2018). Research on whether CAB and CAR denote or work together with coping flexibility in predicting youth adjustment would greatly advance our understanding of the complex processes underlying youth coping with daily hassles. Lastly, the two‐wave longitudinal design and our modest sample size preclude us from disentangling changes in youth adjustment due to within‐ and between‐person processes. Future studies that utilize three or more repeated measures of youth adjustment and model autoregressive latent trajectories are needed to better examine interindividual differences in intraindividual changes.

Despite these limitations, our study contributes to major gaps identified in the literature on coping and stress physiology (Erath & Pettit, 2021) by revealing that a match between more engagement coping behaviors and greater cardiac autonomic capacity to coactivate the parasympathetic and sympathetic branches synergistically predict better social and academic adjustment across transition to middle school. Extending burgeoning work highlighting individual cardiac autonomic functioning as a marker of clinical/psychopathological outcomes (e.g., Berntson et al., 2008), our study is the first to apply CAB/CAR to understand the association between coping with daily hassles and variations in normative developmental outcomes among a community sample of youth. Findings from our study can inform broader efforts within the home and educational settings toward promoting successful adaptation to new peer and academic challenges that accompany the middle school transition. The compatibility between youths’ skillset (i.e., mastery of coping strategies) and biological disposition is critical to facilitate optimal development.

## CONFLICT OF INTEREST

The authors declare no conflict of interest.

## Supporting information




**Table 1A** Descriptive Statistics and Bivariate Correlations for Social and Academic Adjustment Indicators before Standardization
**Table 2A** Descriptive Statistic and Bivariate Correlations for CAB and CAR Reactivity
**Table 3A** Regressions for Coping and Cardiac Autonomic Reactivity Predicting Youth Adjustment

## Data Availability

Please contact the corresponding author regarding the data that support the findings of this study.
