# Supplementary material for: Youth coping and cardiac autonomic functioning: Implications for social and academic adjustment
Source: Dev Psychobiol. 2022 Nov 23;64(8):e22338. doi: 10.1002/dev.22338 (PMC10100426; doi:10.1002/dev.22338)
Supplement: Supplementary file 1 — Table 1A Descriptive Statistics and Bivariate Correlations for Social and Academic Adjustment Indicators before Standardization Table 2A Descriptive Statistic and Bivariate Correlations for CAB and CAR Reactivity Table 3A Regressions for Coping and Cardiac Autonomic Reactivity Predicting Youth Adjustment [file DEV-64-0-s001.docx]

**Online Supplement**

**Table 1A**

*Descriptive Statistics and Bivariate Correlations for Social and Academic Adjustment Indicators before Standardization*

|  | T1/T2 | | | | | Correlations (T1\T2) | | | | | | |
| --- | --- | --- | --- | --- | --- | --- | --- | --- | --- | --- | --- | --- |
| *Social adjustment indicators* | *N* | *M* | *SD* | *Min.* | *Max.* | 1 | 2 | 3 | 4 | 5 | 6 | T1 and T2 |
| 1. Friendship quality | 95/  89 | 3.04/  2.97 | 0.76/  0.67 | 0.79/  1.08 | 4.00/  3.96 | ---- | .14 | .36*** | .24* | .21 | .29* | .69*** |
| 2. Peer acceptance | 99/  89 | 3.23/  3.15 | 0.56/  0.55 | 0.83/  1.50 | 4.00/  4.00 | .26* | ---- | .41*** | .34** | .28* | .29* | .71*** |
| 3. Social skill | 98/  89 | 3.53/  3.74 | 0.70/  0.70 | 1.71/  1.29 | 5.00/  5.00 | .39*** | .57*** | ---- | .27* | .19 | .17 | .63*** |
| 4. Social competence | 78/  76 | 3.10/  3.31 | 0.86/  0.69 | 0.16/  1.12 | 4.00/  4.00 | .29* | .42*** | .53*** | ---- | .32** | .51*** | .64*** |
| 5. Social status | 78/  76 | 5.96/  5.83 | 1.07/  1.09 | 2.33/  2.00 | 7.00/  7.00 | .38*** | .40*** | .40*** | .58*** | ---- | .50*** | .41*** |
| 6. Peer victimization | 78/  76 | 4.60/  4.79 | 0.43/  0.37 | 3.00/  3.09 | 5.00/  5.00 | .28* | .17 | .41*** | .65*** | .39*** | ---- | .48*** |
| *Academic adjustment indicators* | |  |  |  |  | 7 | 8 | 9 | 10 |  |  |  |
| 7. Subject performance | 99/  89 | 3.84/  3.91 | 0.62/  0.68 | 2.40/  1.40 | 5.00/  5.00 | ---- | .34** | .38*** | .32** |  |  | .42*** |
| 8. School and academic  adjustment | 99/  89 | 4.18/  4.15 | 0.66/  0.57 | 1.78/  1.78 | 5.00/  5.00 | .37*** | ---- | .40*** | .44*** |  |  | .27* |
| 9. School engagement | 78/  76 | 3.30/  3.31 | 0.80/  0.67 | 1.00/  1.00 | 4.00/  4.00 | .24* | .46*** | ---- | .78*** |  |  | .61*** |
| 10. Academic functioning | 78/  76 | 4.24/  4.30 | 0.98/  0.87 | 1.33/  1.00 | 5.00/  5.00 | .30** | .47*** | .78*** | ---- |  |  | .59*** |

*Note.* T1/T2 = Time 1/Time 2. The bottom-left half and the top-right half of the correlation matrix represented correlations between adjustment indicators with each other within T1 and T2, respectively. The last column reflected stability of each outcome from T1 to T2 (i.e., correlation between T1 and T2 for the same outcome).

Confirmatory factor analyses were conducted to support the use of a single composite for youth adjustment in each domain at each time point. Results showed that six indicators of social adjustment converged on one factor at each time point (standardized factor loadings ranged from .52-.80 at T1 and .29-.81 at T2), as indicated by satisfactory model fit at T1/T2; *χ^2^* (7) = 9.46/4.00, *p* = .221/780; CFI = .98/1.00, TLI = .95/1.12; RMSEA = .06/.00, [.00/.00, .15/.08]. Similarly, four indicators of academic adjustment converged on one factor at each time point (standardized factor loadings ranged from .49-.77 at T1 and .43-.97 at T2), as indicated by good model fit at T1/T2; *χ^2^* (1) = .53/1.08, *p* = .468/.299; CFI = 1.00/1.00, TLI = 1.05/.99; RMSEA = .00/.03, [.00/.00, .24/.27].

**p* < .05, ***p* < .01, ****p* < .001

Below, we summarized additional results from data check, preliminary analyses, and main model tests for CAB and CAR reactivity. For each domain, missing reactivity scores were primarily due to bad physiological signal during the conversation activity (*n* = 7) and participants not following the administered protocol (*n* = 1). For the peer conversation, reactivity scores were removed for three more dyads because they discussed topic that was unrelated to a peer challenge.

As shown in Table 2A, older youth exhibited lower CAB reactivity in the academic conversation. CAB reactivity in the academic and social conversations were highly correlated, and the same pattern was found for CAR reactivity. However, no significant association emerged for CAB or CAR reactivity with youth coping or adjustment. No difference on reactivity scores was found by study cohort, youth gender, and race/ethnicity. Finally, as shown in Table 3A, neither CAB nor CAR reactivity has significant main or moderating effect on youth adjustment.

**Table 2A**

*Descriptive Statistic and Bivariate Correlations for CAB and CAR Reactivity*

|  |  | 4 | 5 | 6 | 7 |
| --- | --- | --- | --- | --- | --- |
| 1. | T1 age | -.10 | -.08 | -.26* | -.06 |
| 2. | T1 % engagement coping – peer | .10 | -.19 | .08 | -.14 |
| 3. | T1 % engagement coping – academic | -.04 | -.02 | .02 | -.02 |
| 4. | T1 CAB reactivity – peer | ---- |  |  |  |
| 5. | T1 CAR reactivity – peer | -.02 | ---- |  |  |
| 6. | T1 CAB reactivity – academic | .68*** | .12 | ---- |  |
| 7. | T1 CAB reactivity – academic | .03 | .73*** | -.02 | ---- |
| 8. | T1 social adjustment | -.04 | -.01 | -.08 | -.04 |
| 9. | T1 academic adjustment | .12 | -.04 | -.03 | .01 |
| 10. | T2 social adjustment | -.04 | -.03 | -.07 | -.08 |
| 11. | T2 academic adjustment | .03 | -.09 | -.05 | -.12 |
| *M* | | -0.06 | -0.06 | -0.03 | -0.03 |
| *SD* | | 1.34 | 1.46 | 1.41 | 1.39 |
| *Min.* | | -4.20 | -3.58 | -4.32 | -3.80 |
| *Max.* | | 3.65 | 3.66 | 4.03 | 2.81 |
| *N* | | 89 | 89 | 92 | 92 |

*Note.* T1/T2 = Time 1/Time 2. CAB/CAR = cardiac autonomic balance/cardiac autonomic regulation.

**p* < .05. ***p* < .01. ****p* < .001.

**Table 3A**

*Regressions for Coping and Cardiac Autonomic Reactivity Predicting Youth Adjustment*

|  | T2 Social Adjustment | | | | |  | T2 Academic Adjustment | | | | |
| --- | --- | --- | --- | --- | --- | --- | --- | --- | --- | --- | --- |
| *Effects for T1 predictors* | *β* | *B* | *SE* | *p* | *R^2^/ΔR^2^* |  | *β* | *B* | *SE* | *p* | *R^2^/ΔR^2^* |
| *Covariates* |  |  |  |  | 63.6% |  |  |  |  |  | 53.3% |
| Age | .14 | .26 | .12 | .034 |  |  | .19 | .48 | .18 | .009 |  |
| Gender | -.17 | -.11 | .04 | .008 |  |  | -.01 | .00 | .06 | .944 |  |
| African American | .03 | .04 | .08 | .608 |  |  | -.05 | -.09 | .12 | .484 |  |
| Asian | -.04 | -.06 | .09 | .500 |  |  | -.07 | -.14 | .14 | .310 |  |
| European American | -.10 | -.09 | .06 | .128 |  |  | -.03 | -.04 | .08 | .661 |  |
| Hispanic or Latino | -.06 | -.07 | .08 | .348 |  |  | .03 | .04 | .12 | .709 |  |
| Study cohort | -.09 | -.06 | .04 | .147 |  |  | -.20 | -.17 | .06 | .006 |  |
| Adjustment | .75 | .63 | .05 | <.001 |  |  | .67 | .71 | .08 | <.001 |  |
| *Main predictor* |  | | | |  |  |  | | | |  |
| Coping (% engagement) | .15 | 2.22 | .93 | .017 | 0.4% |  | .12 | 1.42 | .88 | .105 | 0.4% |
| *Moderator (separate models)* |  | | | |  |  |  | | | |  |
| CAB reactivity | .07 | .03 | .03 | .316 | 0.6% |  | .08 | .05 | .04 | .281 | 1.0% |
| CAR reactivity | .06 | .02 | .03 | .411 | 0.3% |  | -.06 | -.03 | .04 | .459 | 0% |
| *Interaction (separate models)* |  |  |  |  |  |  |  |  |  |  |  |
| Coping × CAB reactivity | .07 | .88 | .82 | .285 | 0.3% |  | -.01 | -.11 | .60 | .849 | -0.1% |
| Coping × CAR reactivity | .03 | .31 | .80 | .698 | 0% |  | .11 | .99 | .63 | .117 | 2.3% |

*Note*. T1/T2 = Time 1/Time 2. CAB = cardiac autonomic balance (higher scores = PNS > SNS), CAR = cardiac autonomic regulation (higher scores = coactivation). Coping (% engagement) = engagement coping divided by the sum of engagement and disengagement coping. Coping with peer stress and cardiac autonomic reactivity to the peer challenge conversation were entered in models predicting social adjustment. Similarly, coping with academic stress and reactivity to the academic challenge conversation were entered in models predicting academic adjustment. Reactivity scores = conversation $-$ baseline. Coefficients reported from step of entry.
